# Association of coffee intake with bone mineral density: a Mendelian randomization study

**DOI:** 10.3389/fendo.2024.1328748

**Published:** 2024-03-20

**Authors:** Yang Ye, Rui Zhong, Xiao-ming Xiong, Chuan-en Wang

**Affiliations:** ^1^Department of Spinal Surgery, Affiliated Sports Hospital of Chengdu Sport University, Chengdu, China; ^2^School of Sports Medicine and Health, Chengdu Sports University, Chengdu, China

**Keywords:** coffee intake, bone mineral density, Mendelian randomization, causal effect, beneficial

## Abstract

**Background:**

In observational studies, the relationship between coffee intake and bone mineral density (BMD) is contradictory. However, residual confounding tends to bias the results of these studies. Therefore, we used a two-sample Mendelian randomization (MR) approach to further investigate the potential causal relationship between the two.

**Methods:**

Genetic instrumental variables (IVs) associated with coffee intake were derived from genome-wide association studies (GWAS) of the Food Frequency Questionnaire (FFQ) in 428,860 British individuals and matched using phenotypes in PhenoScanner. Summarized data on BMD were obtained from 537,750 participants, including total body BMD (TB-BMD), TB-BMD in five age brackets ≥60, 45-60, 30-45, 15-30, and 0-15 years, and BMD in four body sites: the lumbar spine, the femoral neck, the heel, and the ultradistal forearm. We used inverse variance weighting (IVW) methods as the primary analytical method for causal inference. In addition, several sensitivity analyses (MR-Egger, Weighted median, MR-PRESSO, Cochran’s Q test, and Leave-one-out test) were used to test the robustness of the results.

**Results:**

After Bonferroni correction, Coffee intake has a potential positive correlation with total body BMD (effect estimate [Beta]: 0.198, 95% confidence interval [Cl]: 0.05-0.35, *P*=0.008). In subgroup analyses, coffee intake was potentially positively associated with TB-BMD (45-60, 30-45 years) (Beta: 0.408, 95% Cl: 0.12-0.69, *P*=0.005; Beta: 0.486, 95% Cl: 0.12-0.85, *P*=0.010). In addition, a significant positive correlation with heel BMD was also observed (Beta: 0.173, 95% Cl: 0.08-0.27, *P*=0.002). The results of the sensitivity analysis were generally consistent.

**Conclusion:**

The results of the present study provide genetic evidence for the idea that coffee intake is beneficial for bone density. Further studies are needed to reveal the biological mechanisms and offer solid support for clinical guidelines on osteoporosis prevention.

## Introduction

Osteoporosis (OP) and associated fractures caused by low bone mineral density (BMD) are among the leading causes of death in the elderly. Summarized epidemiologic data show that the average global prevalence of OP ranges from 12.3% (50-59 years) to 49.1% (80-89 years) (1). Aging-related reductions in BMD appear to be difficult to reverse; however, higher peak bone mass (PBM) and lower rates of bone loss have been recognized as effective strategies for preventing/delaying OP (2). Aside from genetic background, an individual’s lifestyle is a key factor in BMD levels (3). Therefore, investigating the relationship between particular dietary habits and BMD is an attractive target.

Coffee is the most popular beverage worldwide. Thus, the health effects of coffee intake have been widely noted and studied. Most of the available evidence suggests that moderate coffee intake reduces the risk of certain chronic diseases and mortality (4, 5). However, the results regarding coffee intake and bone density are controversial. Several cross-sectional and longitudinal studies from different regions have shown that coffee intake is positively associated with BMD and is beneficial to bone health (6–9). Unlike, some cross-sectional studies and meta-analysis results do not support a causal relationship (10, 11). On the contrary, research has also suggested that excessive consumption of caffeinated beverages may have a negative impact on BMD (12). Due to divergent views and insufficient data, recent dietary guidelines for the prevention and treatment of osteoporosis do not include specific recommendations for coffee intake (13).

As a classical method, observational research has made a remarkable contribution to the development of medicine. However, the presence of residual confounding and reverse causation makes it difficult to establish clear underlying associations. In addition, randomized controlled trials (RCTs), which are the gold standard of causal reasoning, are hard to implement to some extent due to ethical constraints and high costs.

Mendelian randomization (MR) is the emerging approach to genetic epidemiology, similar to lifetime RCTs, that strengthens causal inference through genetic variation (14). Concretely, genetic variants reproducibly associated with exposure factors are first identified, and then their aggregate effect on disease outcome is estimated. The main advantage of MR over observational studies is that it greatly reduces the effects of confounders and reverse causation, given that genetic variants are randomly assigned at the time of conception and that pregnancy always precedes disease (15). By using publicly available summarized data, MR does not require additional ethical approvals as well as expensive cost investment compared to RCTs (16). Prior to this, the MR study by Yuan et al. did not suggest a correlation between coffee intake and BMD (17). Benefiting from the continued development of genome-wide association studies (GWAS), we conducted a larger MR study to further explore the potential causal relationship between genetically predicted coffee intake and BMD.

## Methods

### Study design

**Figure 1:**
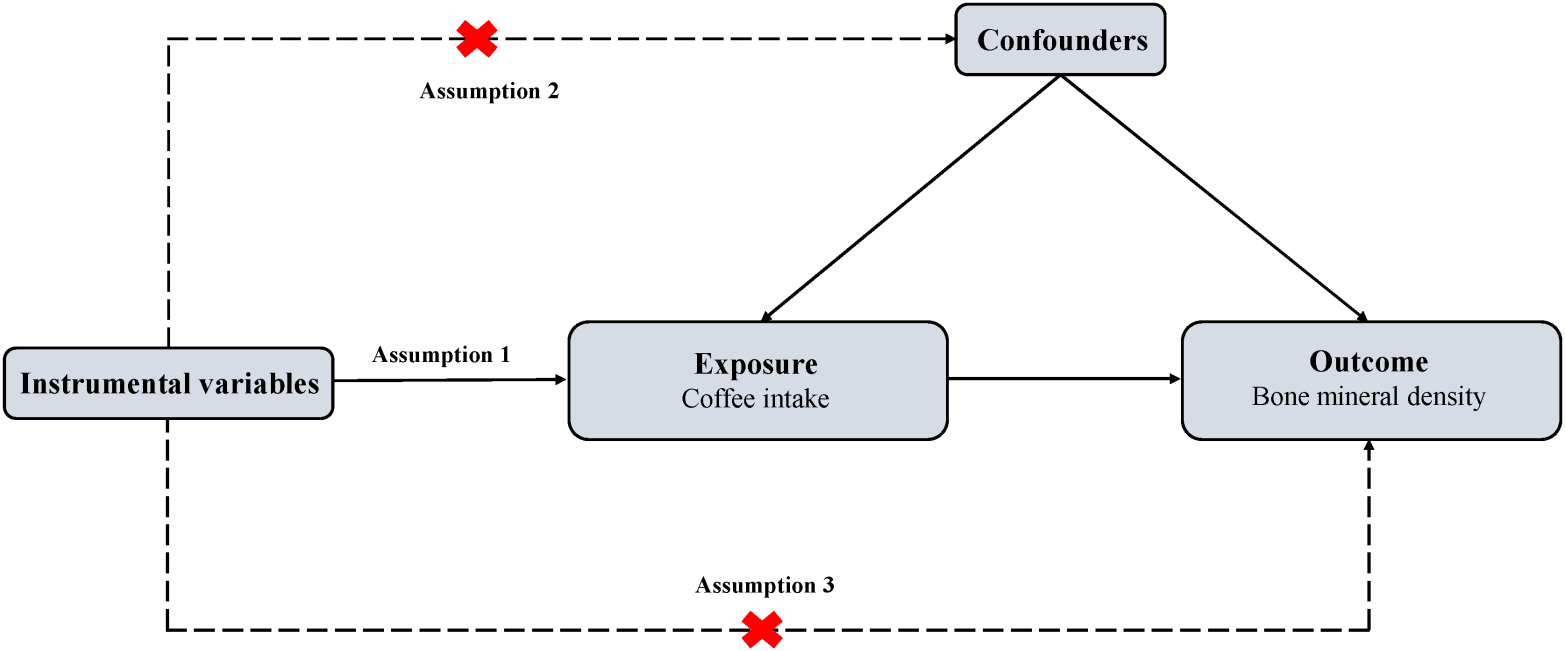
Overview of MR design. MR, Mendelian randomization. Assumption 1: The genetic variants (SNPs) are associated with exposure (coffee intake); Assumption 2: The genetic variants (SNPs) are independent of potential confounders; Assumption 3: The genetic variants (SNPs) affect outcome (bone mineral density) only by the exposure (coffee intake).

We conducted a two-sample MR design with single nucleotide polymorphisms (SNPs) as instrumental variables (IVs) for coffee intake, total body BMD (TB-BMD) was used as the primary outcome. In addition, we performed two subgroup analyses: TB-BMD at five age bracket [including over 60 years (TB-BMD-1), 45-60 years (TB-BMD-2), 30-45 years (TB-BMD-3), 15-30 years (TB-BMD-4) and 0-15 years (TB-BMD-5)] as one group, and the other group with BMD at the site of measurement [including heel (H-BMD), ultradistal forearm (UF-BMD), femoral neck (FN-BMD) and lumbar spine (LS-BMD)]. Summarized data from GWAS on coffee intake and BMD were used to explain causality. To obtain unbiased causal effects, three key assumptions of the approach include 1) SNPs are associated with exposure (coffee intake); 2) SNPs are independent of potential confounders; and 3) SNPs influence outcomes (BMDs) only through exposure (coffee intake) (**Figure 1**). As ethical clearance and consent to participate were obtained for each cohort participating in the GWAS study. Therefore, our study did not require additional review and approval.

### Data sources and instruments

Summary statistics and IVs for coffee intake were obtained from the MRC Integrative Epidemiology Unit (MRC-IEU), and the raw data were the results of the Food Frequency Questionnaire for 428,860 British individuals aged 40 to 69 years (18). We selected all SNPs that independently and strongly (r²<0.001, distance=250kb) predicted cytokines in a genome-wide sense (*P*<5×10^-8^). After aggregation (r^2 =^ 0.001, distance=250kb). Ultimately, a total of 40 SNPs associated with coffee intake were identified for use in the preliminary study. details of the genetic variation of the SNPs are shown in **Supplementary Table S1** of the **Supplementary Material**. Multiple sources of bone density GWAS datasets were available, with the TB-BMD GWAS dataset coming from the GEnetic Factors for OSteoporosis consortium (GEFOS) Consortium meta-analysis (19), which included 66,628 participants who were 86% of European ancestry, 14% of mixed Oceanian ancestry, and 2% African American. GWAS data for heel BMD (H-BMD) (20) and Ultradistal forearm BMD (UF-BMD) (21) were from studies including 426,824 and 21,907 European populations, respectively; whereas GWAS data for lumbar spine BMD (LS-BMD) and femoral neck BMD (FN-BMD) were from another GEFOS study (22), with 32,735 and 28,498 participants of predominantly white British origin. Relevant details are given in **Table 1**.

**Table 1:** Details of the genome-wide association studies and datasets used in this study.

Exposure or outcome	Abbreviations	Sample size	Ancestry	GWAS ID	PMID
Total body bone mineral density	TB-BMD	56,284	European	ebi-a-GCST005348	29304378
Total body bone mineral density (age over 60)	TB-BMD-1	22,504	European	ebi-a-GCST005349	29304378
Total body bone mineral density (age 45-60)	TB-BMD-2	18,805	European	ebi-a-GCST005350	29304378
Total body bone mineral density (age 30-45)	TB-BMD-3	10,062	European	ebi-a-GCST005346	29304378
Total body bone mineral density (age 15-30)	TB-BMD-4	4,180	European	ebi-a-GCST005344	29304378
Total body bone mineral density (age 0-15)	TB-BMD-5	11,807	European	ebi-a-GCST005345	29304378
Heel bone mineral density	H-BMD	426,824	European	ebi-a-GCST006979	30598549
Ultradistal forearm bone mineral density	UF-BMD	21,907	European	ebi-a-GCST90013422	33097703
Femoral neck bone mineral density	FN-BMD	32,735	Mixed	ieu-a-980	26367794
Lumbar spine bone mineral density	LS-BMD	28,498	Mixed	ieu-a-982	26367794
Coffee intake	-	428,860	European	ukb-b-5237	-

GWAS, genome-wide association studies; ID, identity document; PMID, PubMed Unique Identifier.

### Statistical analysis

The Bonferroni method was used to perform a multiple comparison correction to calculate the statistical significance of a *P*<0.005 (0.05/10) according to the number of BMDs. *P*-values between 0.005 and 0.05 were considered suggestive evidence of a potential causal association between the two (23). We performed the statistical analyses in R (version 4.3.1) with TwoSampleMR package (version 0.5.6) and MR-PRESSO package.

The 40 SNPs initially extracted in relation to coffee intake were used as IVs and performed a preliminary MR inverse-variance-weighted (IVW) analysis (**Table 2**). Then, to minimize confounder bias, we used the Phenoscanner database (version 2) (http://www.phenoscanner.medschl.cam.ac.uk/) (24) to examine SNPs individually and exclude SNPs that were either directly associated with BMD or potentially causally associated with BMD for other phenotypes (e.g., smoking and long-term use of hormonal drugs). Besides, to improve the robustness of the results, we performed a multi-stage MR-Pleiotropy RESidual Sum and Outlier (MR-PRESSO) test to identify and eliminate outliers. Finally, We used the IVW method as the main analysis by combining Bate and Standard Errors to assess the correlation and impact of coffee intake and BMD (25). In addition, we performed several sensitivity analyses to verify the robustness of the final results (26). First, we conducted MR-Egger regression to evaluate for directional pleiotropy (27, 28), the intercept close to zero would be regarded as there is no directional pleiotropy. Second, we performed weighted median analysis (29), which has greater robustness to individual genetic instruments with strongly outlying causal evaluations. Third, Cochran’s Q test was employed to estimate the heterogeneity through the evaluates derived from each SNP (30), meanwhile, we calculated *I*² to assess the strength of heterogeneity (31). Fourth, we utilized leave-one-out analysis to estimate whether there is a single SNP that drives the causal association (32).

**Table 2:** Preliminary IVW analysis.

Outcome	No. of SNPs	Beta	SE	*P*-value
TB-BMD	38	0.253	0.098	0.010
TB-BMD-1	38	0.146	0.147	0.320
TB-BMD-2	38	0.322	0.148	0.030
TB-BMD-3	38	0.444	0.180	0.014
TB-BMD-4	38	0.041	0.300	0.891
TB-BMD-5	38	0.175	0.156	0.262
H-BMD	37	0.152	0.066	0.021
UF-BMD	37	0.241	0.135	0.075
FN-BMD	37	0.086	0.090	0.340
LS-BMD	37	0.147	0.127	0.247

Beta, effect estimate; FN-BMD, Femoral neck bone mineral density; H-BMD, Heel bone mineral density; IVW, inverse-variance-weighted; LS-BMD, Lumbar spine bone mineral density; SE, standard error; SNP, single nucleotide polymorphism; TB-BMD, Total body bone mineral density; TB-BMD-1, Total body bone mineral density (age over 60); TB-BMD-2, Total body bone mineral density (age 45-60); TB-BMD-3, Total body bone mineral density (age 30-45); TB-BMD-4, Total body bone mineral density (age 15-30); TB-BMD-5, Total body bone mineral density (age 0-15); UF-BMD, Ultradistal forearm bone mineral density.

## Results

We extracted detailed information about each IV and calculated minor allele frequencies (MAF) and *F*-statistics, which exceeded 10 for all IVs (30-646), indicating the robustness of the IVs. The results of the preliminary MR IVW methodology analysis showed that coffee intake was positively associated with all BMDs in this study and had potential causal associations with TB-BMD, TB-BMD-2, TB-BMD-3, and H-BMD (Beta:0.253, *P*=0.010; Beta:0.322, *P*=0.030; Beta:0.444, *P*=0.014; Beta:0.125, *P*=0.021). Subsequently, we filtered out 2 polyvalent SNPs directly associated with H-BMD and 1 related to current smoking using Phenoscanner (**Table 3**).

**Table 3:** Details of the genetic variants with potential pleiotropy among instrumental variables of coffee intake.

SNP	Pleiotropic trait^*^	*P*-value	PMID
rs1421085	Heel bone mineral density	2.83E-06	UKBB
	Past tobacco smoking	1.45E-06	UKBB
rs76675804	Heel bone mineral density	5.27E-15	UKBB
rs947791	Heel bone mineral density	3.53E-09	UKBB

PMID, PubMed Unique Identifier; SNP, single nucleotide polymorphism; UKBB, UK biobank.

*From the Phenoscanner Database (version 2) (http://www.phenoscanner.medschl.cam.ac.uk, last accessed on Oct. 5th, 2023).

Utilizing the remaining IVs we performed the first MR-PRESSO test. the MR-PRESSO distortion test results showed that there were 1 in TB-BMD and 11 in H-BMD significant outliers and MR-PRESSO global test suggested heterogeneity (TB-BMD: *P*=0.011; H-BMD: *P*<0.001). Besides, no significant outliers were found in other subgroups. After removing the outliers, we performed a second MR-PRESSO test. the MR-PRESSO distortion test suggested the presence of 1 significant outlier in H-BMD, while MR-PRESSO global test suggested the presence of significant heterogeneity (*P*=0.001), whereas no significant abnormality was found in TB-BMD. For H-BMD, we conducted a third MR-PRESSO test. MR-PRESSO distortion test did not suggest significant outliers, but MR-PRESSO global test suggested significant heterogeneity (*P*=0.010, **Tables 4**, **5**).

**Table 4:** Results of IVW and sensitivity analysis.

Outcome	IVW		PLEIO test		Cochran’s Q test		MR-PRESSO global test	
	Beta	*P-*value	Intercept	*P*-value	*I*²	*P-*value	No. of Outliers	*P-*value
TB-BMD	0.254	0.006	<0.001	0.822	41%	0.008	1	0.011
TB-BMD **^a^**	0.198	0.008	-0.0005	0.808	6%	0.362	NA	0.384
TB-BMD-1	0.079	0.576	-0.005	0.224	26%	0.078	NA	0.078
TB-BMD-2	0.408	0.005	0.005	0.311	15%	0.216	NA	0.232
TB-BMD-3	0.486	0.010	0.005	0.451	0%	0.669	NA	0.626
TB-BMD-4	0.026	0.929	0.007	0.485	4%	0.396	NA	0.327
TB-BMD-5	0.124	0.446	<0.001	0.952	0%	0.800	NA	0.824
H-BMD	0.087	0.137	0.002	0.185	85%	<0.001	11	<0.001
H-BMD **^a^**	0.148	0.006	0.002	0.377	56%	<0.001	1	0.001
H-BMD **^b^**	0.173	4.86E-04	0.002	0.574	45%	0.011	NA	0.010
UF-BMD	0.241	0.075	0.004	0.389	16%	0.202	NA	0.192
FN-BMD	0.116	0.220	0.003	0.545	0%	0.552	NA	0.553
LS-BMD	0.147	0.292	0.004	0.781	38%	0.014	NA	0.014

Beta, effect estimate; FN-BMD, Femoral neck bone mineral density; H-BMD, Heel bone mineral density; I²,strength of heterogeneity; IVW, inverse-variance-weighted; LS-BMD, Lumbar spine bone mineral density; MR, Mendelian Randomization; No., number; PLEIO, Pleiotropic Locus Exploration and Interpretation using Optimal test; PRESSO, Pleiotropy RESidual Sum and Outlier; SE, standard error; SNP, single nucleotide polymorphism; TB-BMD, Total body bone mineral density; TB-BMD-1, Total body bone mineral density (age over 60); TB-BMD-2, Total body bone mineral density (age 45-60); TB-BMD-3, Total body bone mineral density (age 30-45); TB-BMD-4, Total body bone mineral density (age 15-30); TB-BMD-5, Total body bone mineral density (age 0-15); UF-BMD, Ultradistal forearm bone mineral density.

**^a^** Results after the first deletion of outliers displayed by the MR-PRESSO analysis.

**^b^** Results after the second deletion of outliers displayed by the MR-PRESSO analysis.

**Table 5:** MR-PRESSO analysis shows outliers.

Outcome	Outliers	EA	Beta	SE	MR-PRESSO global test	
					RSSobs	*P*-value
TB-BMD	rs780093 **^a^**	C	0.013	0.002	7.60e-04	<0.035
H-BMD	rs13054099 **^a^**	C	-0.011	0.002	1.03e-04	<0.034
H-BMD	rs13163336 **^a^**	A	0.0150	0.002	8.19e-05	<0.034
H-BMD	rs1338549 **^a^**	G	-0.009	0.002	5.00e-05	<0.034
H-BMD	rs1527961 **^a^**	C	-0.013	0.002	1.84e-04	<0.034
H-BMD	rs17842490 **^a^**	G	-0.045	0.007	1.15e-04	<0.034
H-BMD	rs1942965 **^a^**	C	-0.009	0.002	1.11e-04	<0.034
H-BMD	rs2189234 **^a^**	G	0.010	0.002	4.46e-05	<0.034
H-BMD	rs2465037 **^a^**	A	-0.011	0.002	3.56e-05	<0.034
H-BMD	rs2472297 **^a^**	T	0.046	0.002	5.18e-05	<0.034
H-BMD	rs2597805 **^a^**	T	0.010	0.002	3.38e-05	<0.034
H-BMD	rs34060476 **^a^**	G	0.018	0.002	5.11e-05	<0.034
H-BMD	rs6063085 **^b^**	C	0.010	0.002	4.58e-05	<0.032

Beta, effect estimate; EA, effect allele; FN-BMD, Femoral neck bone mineral density; H-BMD, Heel bone mineral density; LS-BMD, Lumbar spine bone mineral density; MR, Mendelian Randomization; PRESSO, Pleiotropy RESidual Sum and Outlier; RSSobs, observe the Residual Sum of Squares; SE, standard error; SNP, single nucleotide polymorphism; TB-BMD, Total body bone mineral density; TB-BMD-1, Total body bone mineral density (age over 60); TB-BMD-2, Total body bone mineral density (age 45-60); TB-BMD-3, Total body bone mineral density (age 30-45); TB-BMD-4, Total body bone mineral density (age 15-30); TB-BMD-5, Total body bone mineral density (age 0-15); UF-BMD, Ultradistal forearm bone mineral density.

**^a^** Outliers shown by the first analysis with MR-PRESSO.

**^b^** Outliers shown by the second analysis with MR-PRESSO.

**Figure 2:**
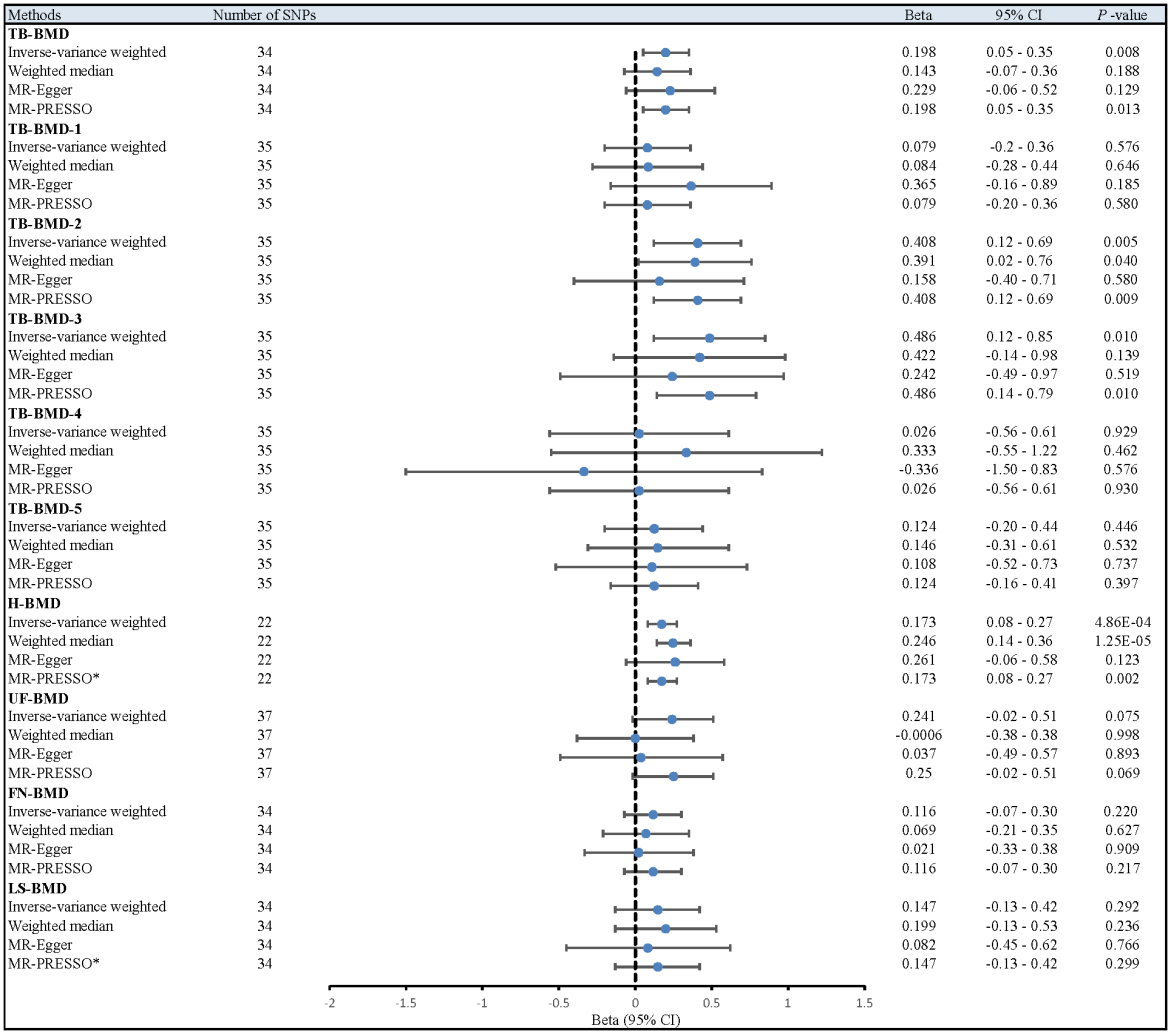
Different MR results for relationship between coffee intake and BMDs. Beta, effect estimate; CI, confidence interval; FN-BMD, Femoral neck bone mineral density; H-BMD, Heel bone mineral density; LS-BMD, Lumbar spine bone mineral density; MR, Mendelian Randomization; PRESSO, Pleiotropy RESidual Sum and Outlier; SNP, single nucleotide polymorphism; TB-BMD, Total body bone mineral density; TB-BMD-1, Total body bone mineral density (age over 60); TB-BMD-2, Total body bone mineral density (age 45-60); TB-BMD-3, Total body bone mineral density (age 30-45); TB-BMD-4, Total body bone mineral density (age 15-30); TB-BMD-5, Total body bone mineral density (age 0-15); UF-BMD, Ultradistal forearm bone mineral density. *MR-PRESSO as the primary analysis.

After multiple corrections, we retained the relatively reliable SNPs as IVs for the final correlation and sensitivity analyses. The MR results for coffee intake and 10 BMDs are shown in **Figure 2**; **Supplementary Table S2**. For H-BMD, *P*-values of both IVW, MR-Egger’s Q-tests, and MR-PRESSO global test were lower than 0.05. Therefore, MR-PRESSO was selected as the main method (33). In addition, MR-PRESSO global test for LS-BMD was lower than 0.05, we also utilized MR-PRESSO as the primary method (33). No evidence of significant heterogeneity was found for other BMDs, so IVW was used as the primary analysis.

**Figure 3:**
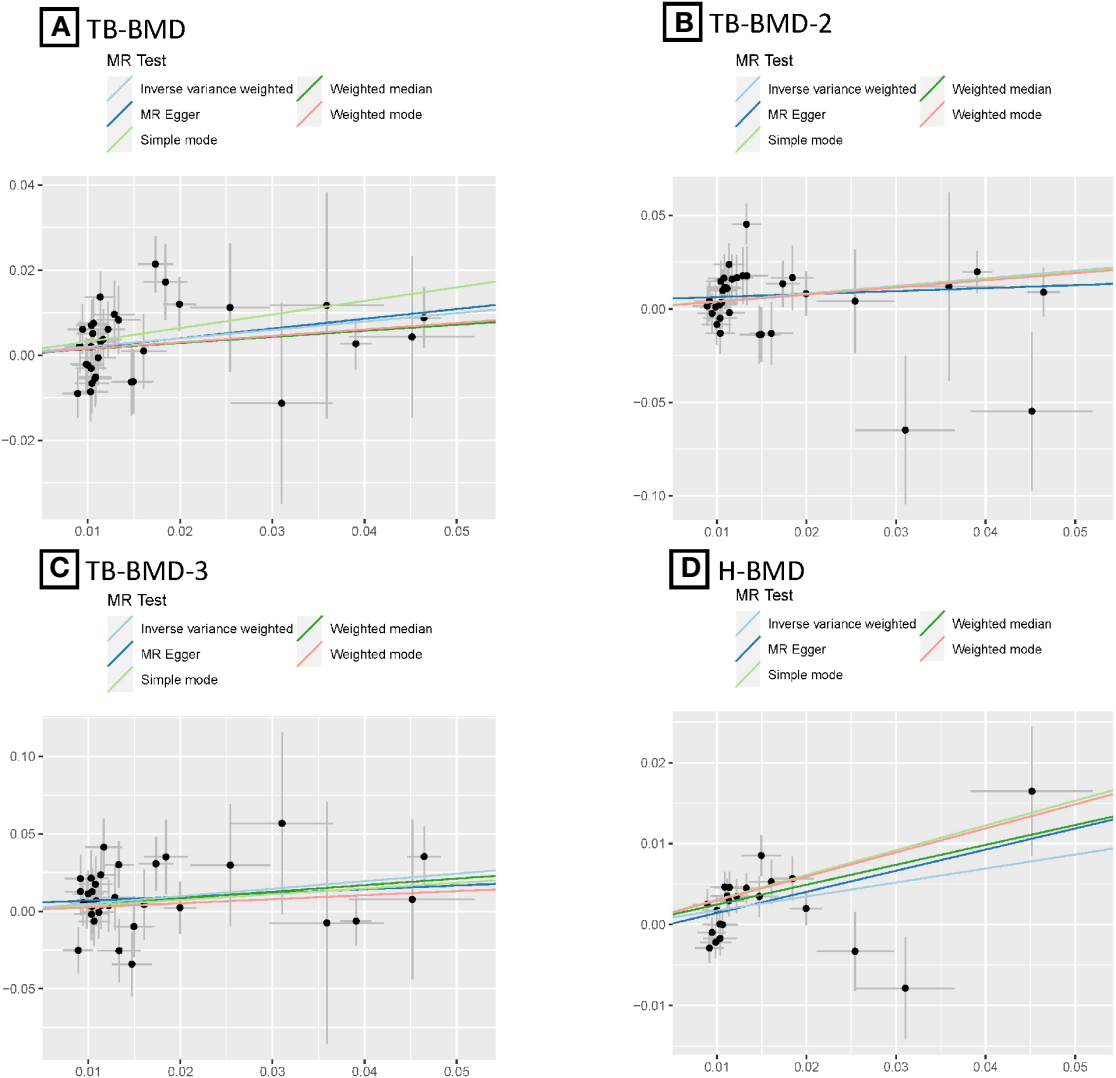
MR scatter plots for relationship of coffee intake with BMDs. **(A)**, Scatter plot of SNPs effects on coffee intake vs TB-BMD; **(B)**, Scatter plot of SNPs effects on coffee intake vs TB-BMD-2; **(C)**, Scatter plot of SNPs effects on coffee intake vs TB-BMD-3; **(D)**, Scatter plot of SNPs effects on coffee intake vs H-BMD. with the slope of each line corresponding to estimated MR effect per method.

**Figure 4:**
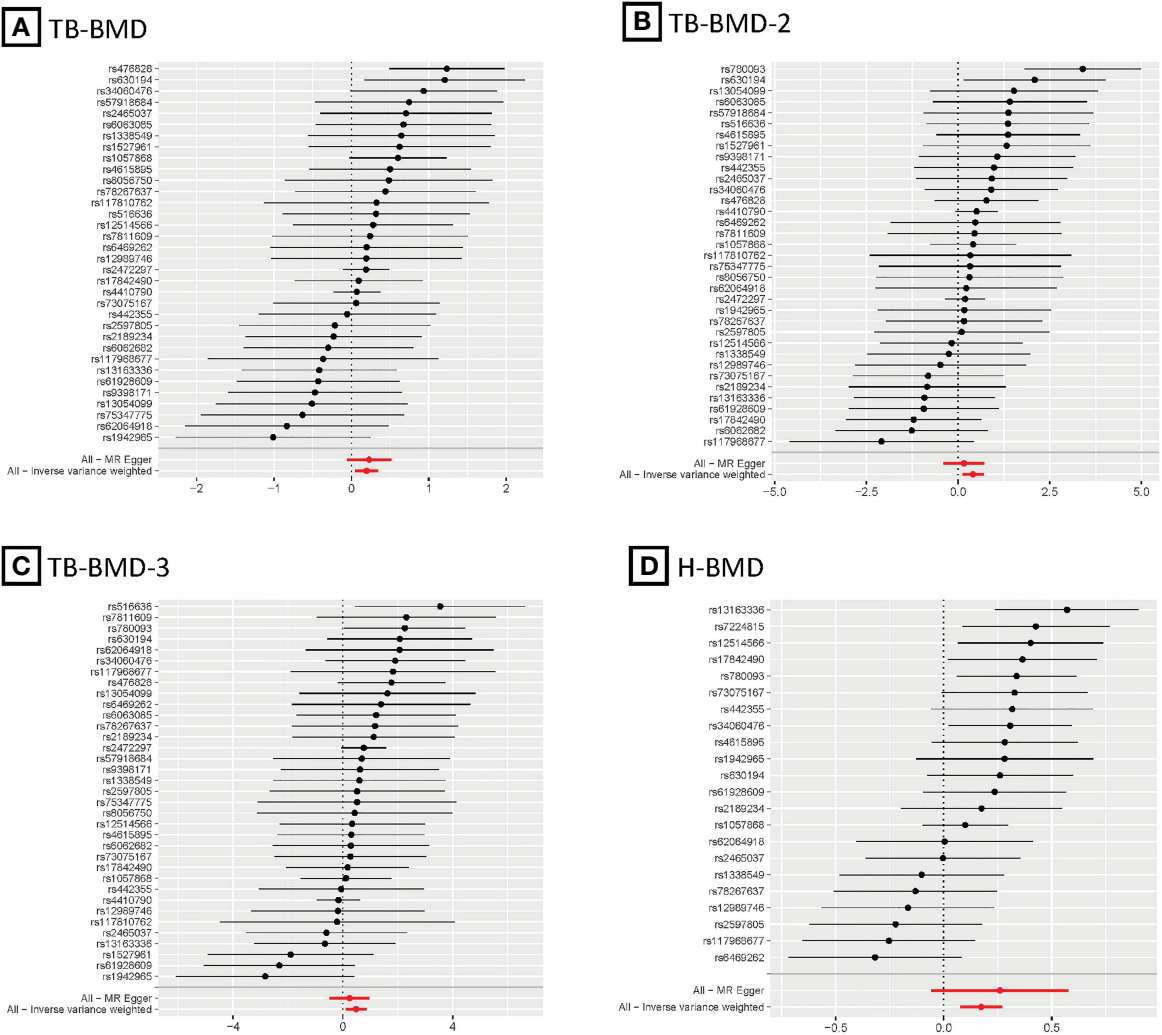
MR forest plots for relationship of coffee intake with BMDs. **(A)**, Forest plot of SNPs effects on coffee intake vs TB-BMD; **(B)**, Forest plot of SNPs effects on coffee intake vs TB-BMD-2; **(C)**, Forest plot of SNPs effects on coffee intake vs TB-BMD-3; **(D)**, Forest plot of SNPs effects on coffee intake vs H-BMD.

**Figure 5:**
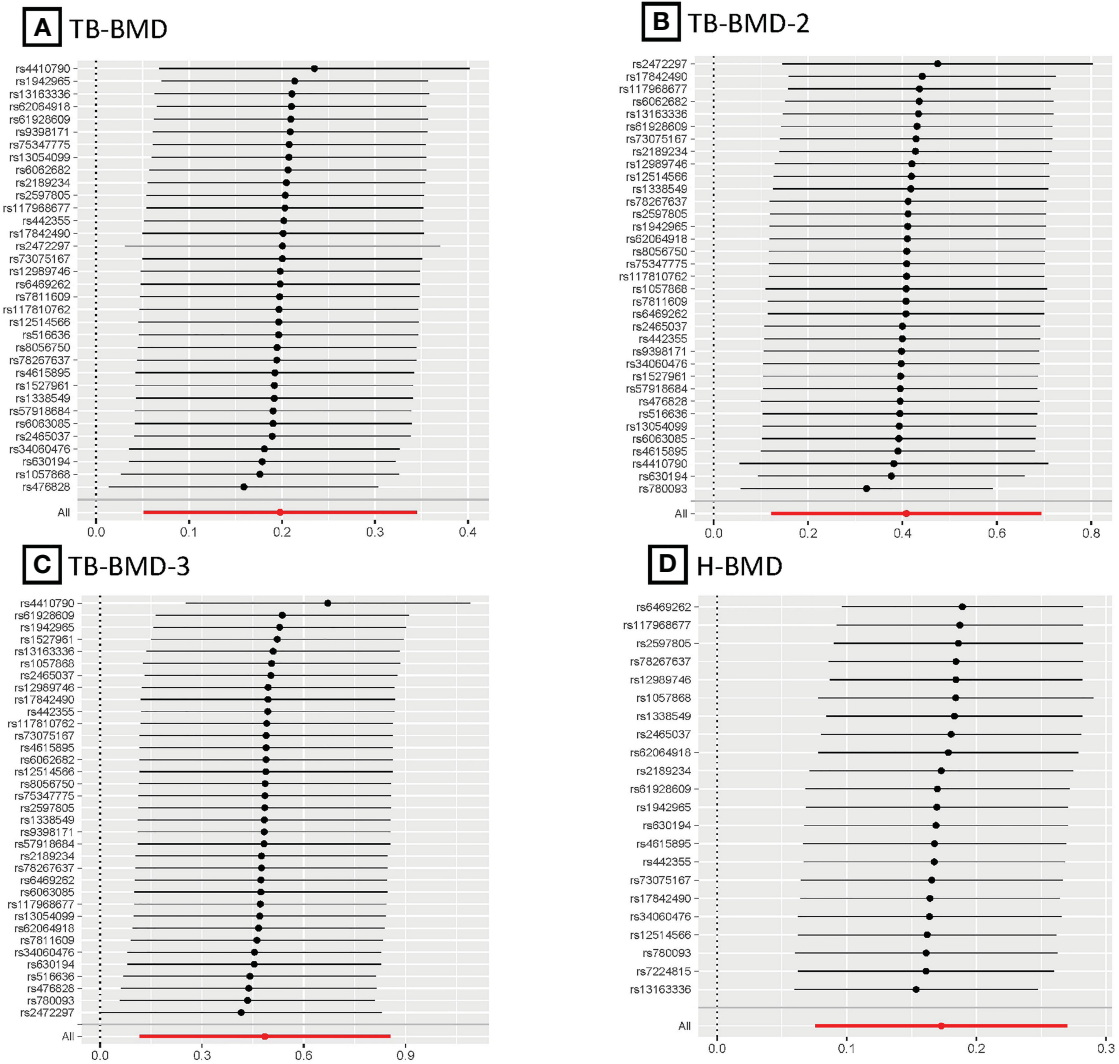
MR leave-one-out plots for relationship of coffee intake with BMDs. **(A)**, Leave-one-out plot of SNPs effects on coffee intake vs TB-BMD; **(B)**, Leave-one-out plot of SNPs effects on coffee intake vs TB-BMD-2; **(C)**, Leave-one-out plot of SNPs effects on coffee intake vs TB-BMD-3; **(D)**, Leave-one-out plot of SNPs effects on coffee intake vs H-BMD.

After the Bonferroni correction, IVW method showed that coffee intake (per 1 SD increase) was potentially associated with a 19.8% increase in TB-BMD (Beta: 0.198, 95% confidence interval [CI]: 0.05-0.35, *P*=0.008). Sensitivity analyses showed similar but not statistically significant trends (weighted median method Bate: 0.143, 95% Cl: -0.07-0.36, *P*=0.188; MR-Egger method (Beta: 0.229, 95% Cl: -0.06-0.52, *P*=0.129). Besides, no evidence of significant heterogeneity in the associations between coffee intake and TB-BMD was found according to *I*² and Cochrane’s Q (*I*²: 6%; *P*=0.362), no directional pleiotropy effect was found for MR-Egger intercept (*P*=0.808). Leave-one-out results showed that, when excluding any of the SNP loci alone, the intercept *P*-value for the association between the two was significant. In subgroup analyses, IVW method showed a potential causal relationship between coffee intake and BT-BMD-2 and TB-BMD-3 (Beta: 0.408, 95% Cl: 0.12-0.69, *P*=0.005; Beta: 0.486, 95% Cl: 0.12-0.85, *P*=0.010), which was similarly demonstrated by the weighted median method for BT-BMD-2 but not BT-BMD-3 (Beta: 0.391, 95% Cl: 0.02-0.76, *P*=0.040; Beta: 0.442, 95% Cl: -0.14-0.98, *P*=0.139), and by MR-Egger, which suggested a similar trend of change but no significant correlation (Beta: 0.158, 95% Cl: -0.40-0.71, *P*=0.580; Beta: 0.242, 95% Cl: -0.49-0.97, *P*=0.519). In addition, *I*² and Cochrane’s Q (*I*²: 15%, *P*=0.216; *I*²: 0%, *P*=0.669) provided evidence of no significant heterogeneity between coffee intake and TB-BMD, and no directed multidirectional effect was found for the MR-Egger intercept (*P*=0.311; *P*=0.451). The results of leave-one-out method were robust as well. Additionally, MR-PRESSO method showed a significant positive correlation between coffee intake and H-BMD (Beta: 0.173, 95% Cl: 0.08-0.27, *P*=0.002), which was validated by weighted median method (Beta: 0.246, 95% Cl: -0.14-0.36, *P*=1.25E-05), and MR-Egger demonstrated a similar, but non-significant, trend (Beta: 0.261, 95% Cl: -0.06-0.58, *P*=0.123). The *I*² and Cochrane’s Q test provided evidence of moderate heterogeneity (*I*²: 45%; *P*=0.011), and no horizontal multidirectional validity was found for MR-Egger intercept (*P*=0.574). Leave-one-out method indicated that the results were not driven by a single SNP. Scatterplots, forest plots, and leave-one-out plots of coffee intake versus TB-BMD, TB-BMD-2, TB-BMD-3, and H-BMD are shown in **Figures 3**–**5**. It can be seen that for adults over the age of 30, consuming more coffee in their daily lives may reduce the rate of bone loss and thus reduce the prevalence of osteoporosis.

## Discussion

In the present study, we performed a large-scale two-sample MR approach to investigate the relationship between genetically predicted coffee intake and BMD. In the major analysis, our evidence suggests a potential positive association between coffee intake and TB-BMD. In the 2 subgroup analyses, coffee intake was similarly found to be potentially positively associated with TB-BMD-2 and TB-BMD-3 and significantly positively related to H-BMD. In addition, although no correlations were found in other age brackets or body parts, all results showed comparable trends.

Our MR results support most previous epidemiologic studies. In a clinical study of Korean postmenopausal women (n=4066), using multivariate logistic regression analysis to adjust for confounders, the prevalence of osteoporosis was found to be 36% lower in a cohort with 2-3 times per day coffee intake (n=998) compared with a cohort with less than 1 time per month (n=872) (9). Besides, the results of another recent cross-sectional study including 7041 U.S. adults (between the ages of 20-49 years) showed that higher coffee intake was significantly and positively associated with LS-BMD (between the ages of 30-39 years) in women (34). In addition, studies from New Zealand, Hong Kong, and Taiwan have produced similar results (6–8).

Caffeine and fenugreek are the most abundant bioactive components in coffee (35). Recent mechanistic studies found that by non-specific antagonism of adenosine receptors, caffeine regulates osteoblast/osteoclast differentiation as well as calcium regulation and alteration of lipid profile (36, 37). In addition, a study identified 12 serum metabolites that were positively associated with coffee intake, and 3 of them, including fenugreek, were related to higher FN/LS-BMD (6). Furthermore, flavonoids in coffee have been found to play direct or indirect beneficial roles in most processes of bone metabolism (36). It promotes bone formation by inducing the expression of genes related to osteoblast differentiation and matrix mineralization (38). Also inhibits bone resorption by suppressing RANKL-induced differentiation of osteoblasts and the expression of histone K and TRAP markers in osteoblast cultures (39, 40). Meanwhile, it reduces inflammatory response, cellular oxidative stress, and free radical production by inhibiting the activity of transcription factor NF-kB. On the other hand, flavonoids have an inhibitory effect on the synthesis of inflammatory mediators (e.g., IL-6, TNF-a), cytokines that normally increase osteoclast activity (41).

The present MR study has several advantages. First, the large sample size provides more credible evidence. We used the largest publicly available GWAS dataset on coffee intake (428,860 individuals) and BMD GWAS dataset (548,094 individuals) and provided new insights into the genetically predicted causal relationship between coffee intake and BMD. Second, we discussed the potential effects of coffee intake on 10 different BMDs, and the different results may offer more detailed reference information for future studies. Third, to reduce the influence of known and unknown confounders on the results, we screened the IVs using the Phenoscanner and MR-PRESSO tests. Fourth, we performed sensitivity analysis using four methods (MR-Egger intercept test, heterogeneity test, MR-PRESSO test, and leave-one-out test) to verify the robustness of the results. Fifth, we select the most appropriate method from the four MR methods (IVW, weighted median, MR-Egger, and MR-PRESSO) to improve the precision of causal estimation.

Some limitations are unavoidable. First, given that coffee intake was a questionnaire survey of individuals aged 40-69 years, possible recall bias would have reduced the accuracy of the results. In further investigations, the results of cross-questionnaires with family members and intra-office can minimize this bias. Second, due to differences in raw materials and preparation processes, individuals also add different flavoring agents according to their preferences. Thus, even equal amounts of coffee intake have different effects on BMD.MR methods can only make preliminary predictions of the correlation between the two by using GWAS summarized data from cohort studies, without being able to account for specific biological mechanisms. Third, even after multiple methods were used to exclude IVs associated with known confounders and/or directly related to the outcome, moderate-strength heterogeneity between coffee intake and H-BMD remained, suggesting that there may be unknown confounders that need to be further explored. Fourth, lifetime rates of change in BMD are typically greater in women than in men, especially in postmenopausal women where the rate of bone loss increases substantially, but the GWAS data used in this analysis were not grouped by sex, so additional analyses could not be performed. Exploring the effect of coffee intake on BMD in gender-specific populations would be an attractive direction to take. Fifth, the inclusion of a limited number of cohort studies of mixed ancestry in the BMD GWAS dataset may have influenced the results of the causal estimates.

## Conclusion

In the current MR study, genetically predicted higher coffee intake was significantly associated with higher H-BMD and positively correlated with T-BMD, T-BMD-2, and T-BMD-3, results suggesting a benefit of coffee intake for osteoporosis prevention. Further studies, such as larger MR studies or clinical trials, are necessary to validate these findings and elucidate the underlying biological mechanisms.

## Data availability statement

The original contributions presented in the study are included in the article/**Supplementary Material**. Further inquiries can be directed to the corresponding author.

## Author contributions

YY: Conceptualization, Data curation, Software, Writing – original draft. C-EW: Project administration, Supervision, Writing – review & editing. RZ: Resources, Visualization, Writing – review & editing. X-MX: Data curation, Software, Validation, Writing – review & editing.
