# Supplementary material for: Association of coffee intake with bone mineral density: a Mendelian randomization study
Source: Front Endocrinol (Lausanne). 2024 Mar 20;15:1328748. doi: 10.3389/fendo.2024.1328748 (PMC10987693; doi:10.3389/fendo.2024.1328748)
Supplement: Supplementary file 1 [file Table_1.docx]

Supplementary Material

**Table S1.** Characteristics of the genetic variants associated with coffee intake. 2

**Table S2.** Effect estimates of the associations between coffee intake and BMD in MR analyses. 4

**Table S1.** Characteristics of the genetic variants associated with coffee intake.

| SNP | Chr | EA | Beta | SE | MAF | *P*-value | *F*-statistic* |
| --- | --- | --- | --- | --- | --- | --- | --- |
| rs10119174 | 9 | C | -0.009 | 0.002 | 0.429 | 1.00E-08 | 32.774 |
| rs1057868 | 7 | T | 0.020 | 0.002 | 0.285 | 5.40E-29 | 124.901 |
| rs117810762 | 10 | A | 0.036 | 0.006 | 0.018 | 6.20E-09 | 33.775 |
| rs117968677 | 15 | A | -0.031 | 0.006 | 0.024 | 1.90E-08 | 31.645 |
| rs12514566 | 5 | A | -0.011 | 0.002 | 0.337 | 2.40E-11 | 44.651 |
| rs12989746 | 2 | T | 0.010 | 0.002 | 0.250 | 2.80E-08 | 30.821 |
| rs13054099 | 22 | C | -0.011 | 0.002 | 0.261 | 4.30E-09 | 34.460 |
| rs13163336 | 5 | A | 0.015 | 0.002 | 0.158 | 1.30E-11 | 45.742 |
| rs1338549 | 6 | G | -0.009 | 0.002 | 0.466 | 5.60E-09 | 33.961 |
| rs13387939 | 2 | A | 0.017 | 0.002 | 0.172 | 9.80E-15 | 59.930 |
| rs1421085 | 16 | C | 0.019 | 0.002 | 0.404 | 1.70E-29 | 127.159 |
| rs1527961 | 2 | C | -0.013 | 0.002 | 0.135 | 1.70E-08 | 31.808 |
| rs17842490 | 22 | G | -0.045 | 0.007 | 0.014 | 3.30E-11 | 44.012 |
| rs1942965 | 18 | C | -0.009 | 0.002 | 0.495 | 3.80E-08 | 30.236 |
| rs2189234 | 4 | G | 0.010 | 0.002 | 0.382 | 1.80E-09 | 36.172 |
| rs2465037 | 6 | A | -0.011 | 0.002 | 0.343 | 4.80E-10 | 38.772 |
| rs2472297 | 15 | T | 0.046 | 0.002 | 0.263 | 1.10E-142 | 646.735 |
| rs2597805 | 4 | T | 0.010 | 0.002 | 0.318 | 2.00E-08 | 31.489 |
| rs34060476 | 7 | G | 0.018 | 0.002 | 0.134 | 7.50E-15 | 60.450 |
| rs4410790 | 7 | C | 0.039 | 0.002 | 0.368 | 1.20E-120 | 545.509 |
| rs442355 | 8 | C | -0.011 | 0.002 | 0.254 | 1.90E-09 | 36.096 |
| rs4615895 | 1 | A | 0.012 | 0.002 | 0.259 | 4.20E-11 | 43.520 |
| rs476828 | 18 | C | 0.017 | 0.002 | 0.237 | 5.60E-20 | 83.751 |
| rs516636 | 1 | A | 0.012 | 0.002 | 0.209 | 4.00E-09 | 34.632 |
| rs56113850 | 19 | C | 0.013 | 0.002 | 0.422 | 8.90E-15 | 60.129 |
| rs57918684 | 17 | A | 0.013 | 0.002 | 0.155 | 8.60E-09 | 33.141 |
| rs6062682 | 20 | T | 0.010 | 0.002 | 0.465 | 2.50E-10 | 40.020 |
| rs6063085 | 20 | C | 0.010 | 0.002 | 0.373 | 4.50E-10 | 38.899 |
| rs61928609 | 12 | C | -0.015 | 0.002 | 0.165 | 1.30E-11 | 45.854 |
| rs62064918 | 17 | T | -0.010 | 0.002 | 0.245 | 4.10E-08 | 30.101 |
| rs630194 | 18 | C | -0.011 | 0.002 | 0.343 | 2.30E-11 | 44.678 |
| rs6469262 | 8 | C | -0.009 | 0.002 | 0.435 | 1.90E-08 | 31.576 |
| rs7224815 | 17 | T | -0.011 | 0.002 | 0.408 | 3.70E-11 | 43.765 |
| rs73075167 | 7 | T | -0.016 | 0.002 | 0.129 | 5.00E-11 | 43.191 |
| rs75347775 | 19 | A | 0.010 | 0.002 | 0.245 | 2.70E-08 | 30.933 |
| rs780093 | 2 | C | 0.013 | 0.002 | 0.384 | 1.00E-15 | 64.366 |
| rs7811609 | 7 | T | 0.009 | 0.002 | 0.375 | 4.00E-08 | 30.137 |
| rs78267637 | 8 | G | -0.025 | 0.004 | 0.038 | 3.90E-09 | 34.695 |
| rs8056750 | 16 | T | 0.011 | 0.002 | 0.359 | 1.30E-09 | 36.776 |
| rs9398171 | 6 | T | 0.011 | 0.002 | 0.289 | 1.10E-09 | 37.210 |

**Abbreviations:** Beta, effect estimate; Chr, chromosome; EA, effect allele; MAF, minor allele frequency; SE, standard error; SNP, single nucleotide polymorphism.

**F*-statistic = Beta² / SE².

**Table S2.** Effect estimates of the associations between coffee intake and BMD in MR analyses.

| Methods | No. of SNPs | Beta | 95% CI | *P-*value |  |
| --- | --- | --- | --- | --- | --- |
| TB-BMD |  |  |  |  |  |
| Inverse-variance weighted | 34 | 0.198 | 0.05 – 0.35 | 0.008 |  |
| Weighted median | 34 | 0.143 | -0.07 - 0.36 | 0.188 |  |
| MR-Egger | 34 | 0.229 | -0.06 - 0.52 | 0.129 |  |
| MR-PRESSO | | 34 | 0.198 | 0.05 - 0.35 | 0.013 |
| TB-BMD-1 |  |  |  |  |  |
| Inverse-variance weighted | 35 | 0.079 | -0.20 - 0.36 | 0.576 |  |
| Weighted median | 35 | 0.084 | -0.28 - 0.44 | 0.646 |  |
| MR-Egger | 35 | 0.365 | -0.16 - 0.89 | 0.185 |  |
| MR-PRESSO | 35 | 0.079 | -0.20 - 0.36 | 0.580 |  |
| TB-BMD-2 |  |  |  |  |  |
| Inverse-variance weighted | 35 | 0.408 | 0.12 - 0.69 | 0.005 |  |
| Weighted median | 35 | 0.391 | 0.02 - 0.76 | 0.040 |  |
| MR-Egger | 35 | 0.158 | -0.40 - 0.71 | 0.580 |  |
| MR-PRESSO | 35 | 0.408 | 0.12 - 0.69 | 0.009 |  |
| TB-BMD-3 |  |  |  |  |  |
| Inverse-variance weighted | 35 | 0.486 | 0.12 - 0.85 | 0.010 |  |
| Weighted median | 35 | 0.422 | -0.14 - 0.98 | 0.139 |  |
| MR-Egger | 35 | 0.242 | -0.49 - 0.97 | 0.519 |  |
| MR-PRESSO | 35 | 0.486 | 0.14 - 0.79 | 0.010 |  |
| TB-BMD-4 |  |  |  |  |  |
| Inverse-variance weighted | 35 | 0.026 | -0.56 - 0.61 | 0.929 |  |
| Weighted median | 35 | 0.333 | -0.55 - 1.22 | 0.462 |  |
| MR-Egger | 35 | -0.336 | -1.50 - 0.83 | 0.576 |  |
| MR-PRESSO | 35 | 0.026 | -0.56 - 0.61 | 0.930 |  |
| TB-BMD-5 |  |  |  |  |  |
| Inverse-variance weighted | 35 | 0.124 | -0.20 - 0.44 | 0.446 |  |
| Weighted median | 35 | 0.146 | -0.31 - 0.61 | 0.532 |  |
| MR-Egger | 35 | 0.108 | -0.52 - 0.73 | 0.737 |  |
| MR-PRESSO | 35 | 0.124 | -0.16 - 0.41 | 0.397 |  |
| H-BMD |  |  |  |  |  |
| Inverse-variance weighted | 22 | 0.173 | 0.08 - 0.27 | 4.86E-04 |  |
| Weighted median | 22 | 0.246 | 0.14 - 0.36 | 1.25E-05 |  |
| MR-Egger | 22 | 0.261 | -0.06 - 0.58 | 0.123 |  |
| MR-PRESSO* | 22 | 0.173 | 0.08 - 0.27 | 0.002 |  |
| UF-BMD |  |  |  |  |  |
| Inverse-variance weighted | 37 | 0.241 | -0.02 - 0.51 | 0.075 |  |
| Weighted median | 37 | -0.0006 | -0.38 - 0.38 | 0.998 |  |
| MR-Egger | 37 | 0.037 | -0.49 - 0.57 | 0.893 |  |
| MR-PRESSO | | 37 | 0.250 | -0.02 - 0.51 | 0.069 |
| FN-BMD |  |  |  |  |  |
| Inverse-variance weighted | 34 | 0.116 | -0.07 - 0.30 | 0.220 |  |
| Weighted median | 34 | 0.069 | -0.21 - 0.35 | 0.627 |  |
| MR-Egger | 34 | 0.021 | -0.33 - 0.38 | 0.909 |  |
| MR-PRESSO | | 34 | 0.116 | -0.07 - 0.30 | 0.217 |
| LS-BMD |  |  |  |  |  |
| Inverse-variance weighted | 34 | 0.147 | -0.13 - 0.42 | 0.292 |  |
| Weighted median | 34 | 0.199 | -0.13 - 0.53 | 0.236 |  |
| MR-Egger | 34 | 0.082 | -0.45 - 0.62 | 0.766 |  |
| MR-PRESSO* | | 34 | 0.147 | -0.13 - 0.42 | 0.299 |

**Abbreviations:** Beta, effect estimate; CI, confidence interval; FN-BMD, Femoral neck bone mineral density; H-BMD, Heel bone mineral density; LS-BMD, Lumbar spine bone mineral density; MR, Mendelian Randomization; No., number; PRESSO, Pleiotropy RESidual Sum and Outlier; SNP, single nucleotide polymorphism; TB-BMD, Total body bone mineral density; TB-BMD-1, Total body bone mineral density (age over 60); TB-BMD-2, Total body bone mineral density (age 45-60); TB-BMD-3, Total body bone mineral density (age 30-45); TB-BMD-4, Total body bone mineral density (age 15-30); TB-BMD-5, Total body bone mineral density (age 0-15); UF-BMD, Ultradistal forearm bone mineral density.

* MR-PRESSO as the primary analysis.
